# A cross-study gene set enrichment analysis identifies critical pathways in endometriosis

**DOI:** 10.1186/1477-7827-7-94

**Published:** 2009-09-08

**Authors:** Hongbo Zhao, Qishan Wang, Chunyan Bai, Kan He, Yuchun Pan

**Affiliations:** 1 School of Agriculture and Biology, Shanghai Jiao Tong University, Shanghai, PR China; 2 Shanghai Key Lab for Veterinary Biotechnology, Shanghai, 200240, PR China

## Abstract

**Background:**

Endometriosis is an enigmatic disease. Gene expression profiling of endometriosis has been used in several studies, but few studies went further to classify subtypes of endometriosis based on expression patterns and to identify possible pathways involved in endometriosis. Some of the observed pathways are more inconsistent between the studies, and these candidate pathways presumably only represent a fraction of the pathways involved in endometriosis.

**Methods:**

We applied a standardised microarray preprocessing and gene set enrichment analysis to six independent studies, and demonstrated increased concordance between these gene datasets.

**Results:**

We find 16 up-regulated and 19 down-regulated pathways common in ovarian endometriosis data sets, 22 up-regulated and one down-regulated pathway common in peritoneal endometriosis data sets. Among them, 12 up-regulated and 1 down-regulated were found consistent between ovarian and peritoneal endometriosis. The main canonical pathways identified are related to immunological and inflammatory disease. Early secretory phase has the most over-represented pathways in the three uterine cycle phases. There are no overlapping significant pathways between the dataset from human endometrial endothelial cells and the datasets from ovarian endometriosis which used whole tissues.

**Conclusion:**

The study of complex diseases through pathway analysis is able to highlight genes weakly connected to the phenotype which may be difficult to detect by using classical univariate statistics. By standardised microarray preprocessing and GSEA, we have increased the concordance in identifying many biological mechanisms involved in endometriosis. The identified gene pathways will shed light on the understanding of endometriosis and promote the development of novel therapies.

## Background

Endometriosis is defined as the presence of endometrium-like tissue in sites outside the uterine cavity and occurs in 6-10% of women in the general population [1]. The main clinical features are chronic pelvic pain, pain during intercourse, and infertility [2]. As cellular and molecular mechanisms involved in endometriosis are still uncovered, the classification of this disease evolved from a local disorder to a complex, chronic systemic disease [3]. Despite extensive researches, the etiology of endometriosis remains obscure. Gene expression profiling has been used in several studies of endometriosis, in which from a few to hundreds differentially expressed genes were identified [4-17]. For previously identified genes, their roles in the pathogenesis of endometriosis are further discussed. But it is hard to interpret individual genes on a list with many significant genes.

A common challenge in the analysis of genome wide expression no longer lies in obtaining gene expression profiles, but rather in interpreting the results to gain insights into biological mechanisms [18]. Pathway analysis of microarray data evaluates gene expression profiles of a priori defined biological pathways in association with a phenotype of interest. Recently gene expression patterns were further used in the classification of subtypes of endometriosis as well as in the identification of the pathways involved in endometriosis [4,13-16]. So far the observed pathways were discordant between the studies that suggest that these previously identified pathways only represent a fraction of the pathways involved in endometriosis.

Currently the most well-known and widely used approach to gene set analysis, the Gene Set Enrichment Analysis (GSEA) method was introduced by Mootha et al. [19], which was used to identify pre-defined gene sets which exhibited significant differences in expression between samples from normal and patients. The methodology was subsequently refined by Subramanian et al. [18]. The algorithms calculate the statistical significance of the expression changes across groups or pathways rather than individual gene, thus allowing identification of groups or pathways most strongly affected by the observed expression changes. The analysis based on a group of relevant genes instead of on an individual gene increases the likelihood for investigators to identify the critical functional processes under the biological phenomena under study. GSEA is likely to be more powerful than conventional single-gene methods in the study of complex diseases in which many genes make subtle contributions [20].

In a single data set the GSEA will generally not result in significant findings beyond major pathways. Here we will use standardised microarray preprocessing and GSEA with comprehensive expression profiles in an attempt to find greater data convergence and provide a systematic insight into the pathways altered during endometriosis pathogenesis.

## Methods

### Datasets

We searched GEO , and ArrayExpress  for the gene expression profiling studies related to endometriosis disease. Data were included in our re-analysis if they met the following conditions: 1) the data is in genome-wide; 2) comparison was conducted between endometriosis patients and controls; 3) complete microarray raw or normalized data are available.

Finally six public gene expression data sets were involved in our study, which assessed endometriosis transcripts on a genome-wide basis. In data set GSE7307, total 677 samples from more than 90 distinct tissue types were processed, but only the profiles related to endometriosis and eutopic endometrium were considered here. The data generated from human endometrial endothelial cells by Sha et al. [4] were also included in our combined re-analysis to compare with the whole endometriosis tissues data sets. The related information about these datasets, such as the microarray platform, sample type, sample size, is listed in Table 1.

**Table 1:** Characteristics of datasets included in the studies.

**First Author** **or Contributor**	**Chip**	**GEO Accession**	**Experimental design**	**Classification**	**Probes**	**Number of samples**	
						**Disease**	**Normal**
Sha [4]	U133 PLUS 2.0	GSE7846	unpaired, HEECS	ovarian	54K	5	5
Burney [14]	U133 PLUS 2.0	GSE6364	unpaired, tissues	Ovarian, peritoneal, rectovaginal	54K	21	16
Eyster [15]	CodeLink	GSE5108	paired, tissues	Ovarian, peritoneal	55K	6/5	6/5
Hever [16]	U133 PLUS 2.0	GSE7305	paired, tissues	ovarian	54K	10	10
Roth	U133 PLUS 2.0	GSE7307	unpaired, tissues	ovarian	54K	18	23
Hull [17]	U133A	GSE11691	paired, tissues	peritoneal	22K	9	9

paried: compare eutopic endometrium to ectopic endometrium from the same patients with entire endometrial tissue.

unparied: compare eutopic endometrium from women with endometriosis to eutopic endometrium from women without endometriosis.

HEECS: human endometrial endothelial cells samples.

### Data Preprocessing

Data preprocessing was performed using software packages developed in version 2.4.0 of Bioconductor [21] and R version 2.9.0 [22]. Each Affymetrix dataset was background adjusted, normalized and log2 probe-set intensities calculated using the Robust Multichip Averaging (RMA) algorithm in affy package [23,24], and the Codelink arrays normalizations performed in GSE5108 were retained. Genes which cannot be mapped to any KEGG pathway identified were excluded from the further analysis. The interquartile range (IQR) was used as a measure of variability. From the resulting distribution of IQR values for all genes we set a cut-off so as to exclude values under 0.5. When multiple probe sets target one gene, the probe set with largest variability was kept. Pathway analysis was performed separately in each data set.

### Gene set enrichment analysis of pathways

GSEA implemented in the Category package (version 2.10.1 [25]). The goal of GSEA is to determine whether the members of a gene set *S *randomly distributed throughout the entire reference gene list *L *or are primarily found at the top or bottom. One of the advantages of GSEA is the relative robustness to noise and outliers in the data. The gene sets represented by less than 10 genes were excluded. The t-statistic mean of the genes was computed in each pathway. Using a permutation test with 1000 times, the significantly changed pathways were identified with p-value ≤ 0.05.

## Results

For the studies which used multiple locations (ovarian and peritoneal) or uterine cycle phases (proliferative to secretory) of endometriosis, each type or phase was treated as a separate data set. These six studies provided 9 case-control data sets including 74 endometriosis cases and 74 controls. Common GSEA method was applied to the 9 datasets. For individual analysis, we obtained the significant pathways and the genes included [See Additional file 1, 2, 3 and 4]. The analysis results were summarised in table 2. We postulated that the pathways and genes that appear consistently as different expressed in multiple studies are more likely to be important in endometriosis. To look for such convergence we compared the GSEA results.

**Table 2:** Summary of each data set used in the re-analysis and the number of differentially expressed pathways.

**Studies**	**Number of patients**	**Number of controls**	**Number of genes after preprocessing**	**Number of pathways have genes ≥ 10**	**Up-regulated pathways**	**Down-regulated pathways**
Ovarian Data Sets						
Eyster	6	6	3105	171	24	31
Hever	10	10	1930	139	56	59
Roth	18	23	2575	154	36	44
Peritoneal Data Sets						
Eyster	5	5	3084	171	57	11
Hull	9	9	1255	113	29	7
Uterine cycle Data Sets						
Burney_Pro	6	5	1900	138	4	3
Burney_ES	6	3	1471	122	12	21
Burney_MS	9	8	952	93	0	22
Human endometrial endothelial cells Data Set						
Sha	5	5	2324	153	44	1

Pro, Proliferative; ES, early secretory; MS, midsecretory Proliferative

### Common significant pathways in ovarian endometriosis

**Figure 1:**
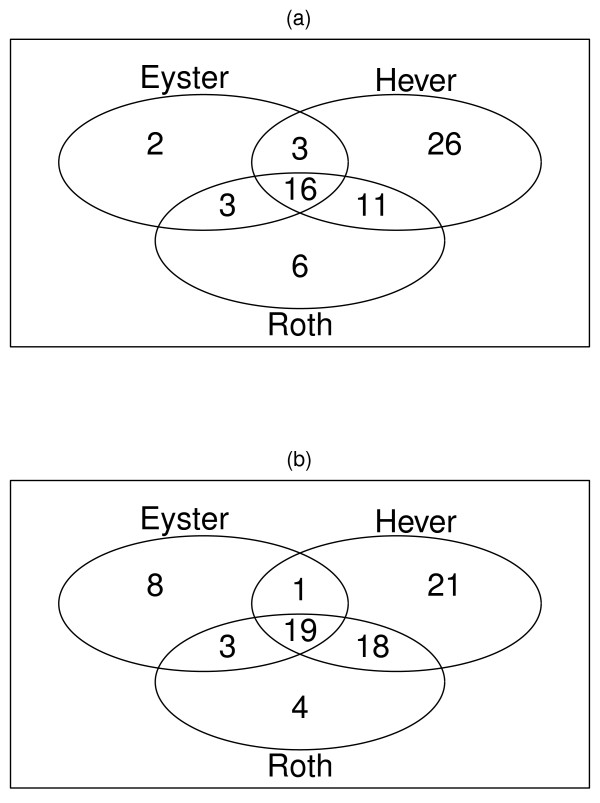
**Significant pathways identified and overlap between the 3 ovarian endometriosis data sets**. For each data set, GSEA implanted to generate p-values for each pathway and using a permutation test with 1000 times, obtained the significant pathways with p-value cut-off of ≤ 0.05. (a) GSEA detected 24 (Eyster data set), 56 (Hever data set) and 36 (Roth data set) up-regulated pathways and 16 are common. (b) GSEA detected 31 (Eyster data set), 59 (Hever data set) and 44(Roth data set) down-regulated pathways and 19 are common.

Endometriosis is most commonly localized in the ovaries. We first compared the ovarian endometriosis data sets for their convergence and reproducibility. The results for the ovarian endometriosis data sets were presented in Table 3. Based on the permutation p-values, the GSEA reported 24 & 31 (Eyster data set), 56 & 59 (Hever data set), and 36 & 44 (Roth data set) up- and down- regulated pathways. The overlaps among them are presented in Figure 1, with 16 up-regulated and 19 down-regulated pathway detected in common. Among the up-regulated list, the main canonical pathways affected are related to immunological and inflammatory diseases including Asthma, Autoimmune thyroid disease, Systemic lupus erythematosus, Allograft rejection, Graft-versus-host disease and Type I diabetes mellitus. Cytokine-cytokine receptor interaction and Cell adhesion molecules (CAMs) belongs to signaling molecules and interaction. PPAR signaling pathway is part of endocrine system. The most down-regulated pathways are metabolism and repair related pathways. And we extracted the collective genes in each pathway from the 3 data sets [See Additional file 5].

**Table 3:** Common pathway categories identified by GSEA of the 3 ovarian endometriosis datasets

**Pathway name**	**Classification**	**Genes**
Up-regulation		
PPAR signaling pathway	Endocrine System	26
Cytokine-cytokine receptor interaction	Signaling Molecules and Interaction	84
Cell adhesion molecules (CAMs)	Signaling Molecules and Interaction	68
Complement and coagulation cascades	Immune System	32
Toll-like receptor signaling pathway	Immune System	37
Hematopoietic cell lineage	Immune System	36
Asthma	Immune Disorders	12
Autoimmune thyroid disease	Immune Disorders	16
Systemic lupus erythematosus	Immune Disorders	29
Allograft rejection	Immune Disorders	16
Graft-versus-host disease	Immune Disorders	17
Arachidonic acid metabolism	Lipid Metabolism	19
Metabolism of xenobiotics by cytochrome P450	Xenobiotics Biodegradation and Metabolism	19
Drug metabolism - cytochrome P450	Xenobiotics Biodegradation and Metabolism	20
Type I diabetes mellitus	Metabolic Disorders	19
Olfactory transduction	Sensory System	4
		
Down-regulation		
Fatty acid metabolism	Lipid Metabolism	21
Androgen and estrogen metabolism	Lipid Metabolism	5
Sphingolipid metabolism	Lipid Metabolism	18
Oxidative phosphorylation	Energy Metabolism	44
Citrate cycle (TCA cycle)	Carbohydrate Metabolism	13
Butanoate metabolism	Carbohydrate Metabolism	12
Pyrimidine metabolism	Nucleotide Metabolism	36
Glycosylphosphatidylinositol(GPI)-anchor biosynthesis	Glycan Biosynthesis and Metabolism	10
Lysine degradation	Amino Acid Metabolism	19
Folate biosynthesis	Metabolism of Cofactors and Vitamins	10
DNA polymerase	Replication and Repair	22
Base excision repair	Replication and Repair	16
Nucleotide excision repair	Replication and Repair	19
Mismatch repair	Replication and Repair	13
Homologous recombination	Replication and Repair	10
Cell cycle	Cell Growth and Death	57
Thyroid cancer	Cancers	15
Proteasome	Folding, Sorting and Degradation	13
Ubiquitin mediated proteolysis	Folding, Sorting and Degradation	50

Genes: Number of common genes included in the pathway.

### Common significant pathways in peritoneal endometriosis

**Figure 2:**
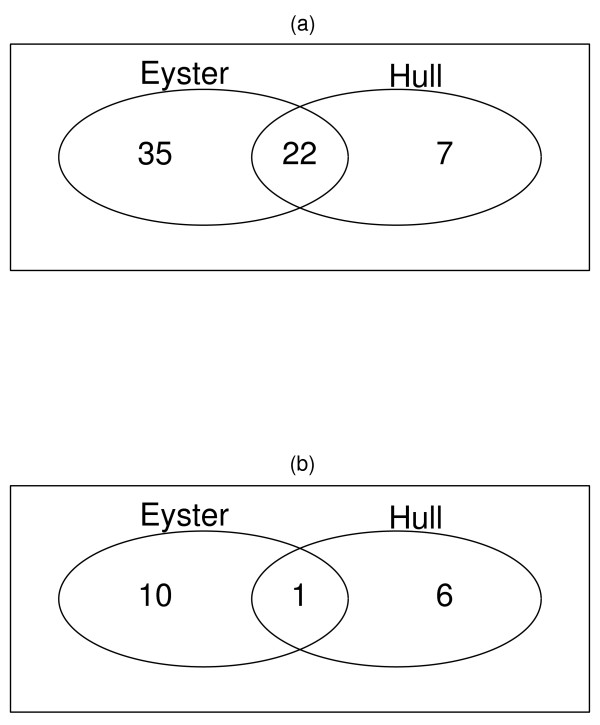
**Significant pathways identified and overlap between the 2 peritoneal endometriosis data sets**. For each data set, GSEA implanted to generate p-values for each pathway and using a permutation test with 1000 times, obtained the significant pathways with p-value cut-off of ≤ 0.05. (a) GSEA detected 57 (Eyster data set) and 29 (Hull data set) up-regulated pathways and 22 are common. (b) GSEA detected 11 (Eyster data set) and 7 (Hull data set) down-regulated pathways and 1 is common.

We also analyzed the data sets from peritoneal endometriosis which is another important endometriosis type. 23 pathways were commonly significantly regulated in the two peritoneal endometriosis data sets, summarised in table 4 and Figure 2. Of these 23 pathways, 12 up- and one down- regulation pathways were also identified from the three ovarian endometriosis data sets. The significances of four pathways (Drug metabolism - cytochrome P450, Metabolism of xenobiotics by cytochrome P450, Olfactory transduction, Toll-like receptor signaling pathway) were found from the data of Eyster et al. [15], but not in the data of Hull et al. [17]. Collective genes in each pathway were extracted [See Additional file 6].

**Table 4:** Common pathway categories identified by GSEA of the 2 peritoneal endometriosis datasets

**Pathway name**	**Classification**	**Genes**
Up-regulation		
Cell Communication	Cell Communication	42
Focal adhesion	Cell Communication	91
Regulation of actin cytoskeleton	Cell Motility	61
PPAR signaling pathway	Endocrine System	19
Calcium signaling pathway	Signal Transduction	41
VEGF signaling pathway	Signal Transduction	25
Cytokine-cytokine receptor interaction	Signaling Molecules and Interaction	58
Neuroactive ligand-receptor interactio	Signaling Molecules and Interaction	20
ECM-receptor interaction	Signaling Molecules and Interaction	46
Cell adhesion molecules (CAMs)	Signaling Molecules and Interaction	49
Complement and coagulation cascades	Immune System	29
Hematopoietic cell lineage	Immune System	29
Leukocyte transendothelial migration	Immune System	44
Asthma	Immune Disorders	7
Autoimmune thyroid disease	Immune Disorders	12
Systemic lupus erythematosus	Immune Disorders	20
Allograft rejection	Immune Disorders	12
Graft-versus-host disease	Immune Disorders	15
Arachidonic acid metabolism	Lipid Metabolism	16
Type I diabetes mellitus	Metabolic Disorders	15
Pancreatic cancer	Cancers	24
Glioma	Cancers	21
		
Down-regulation		
Sphingolipid metabolism	Lipid Metabolism	13

Genes: Number of common genes included in the pathway.

### Differential pathways between the timing of the uterine cycle

**Figure 3:**
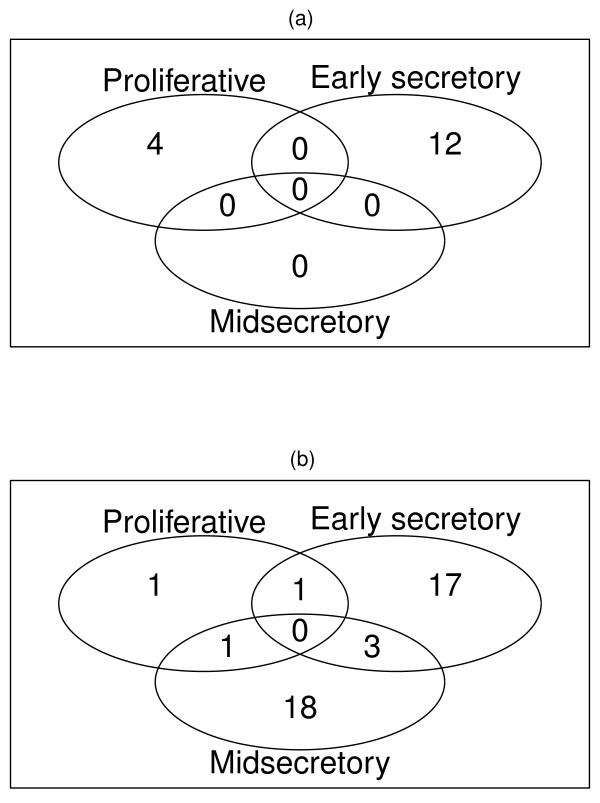
**Significant pathways identified and overlap between the phases of uterine cycle**. For each phase, GSEA implanted to generate p-values for each pathway and using a permutation test with 1000 times, obtained the significant pathways with p-value cut-off of ≤ 0.05. (a) GSEA detected 4 (Proliferative phase), 12 (Early secretory phase) and 0 (Mid secretory phase) up-regulated pathways and no overlap. (a) GSEA detected 3 (Proliferative phase), 21 (Early secretory phase) and 22 (Mid secretory phase) down-regulated pathways and the overlap is very low.

Burney et al. conducted global gene expression analysis of endometrium from women with and without moderate/severe stage endometriosis and compared the gene expression signatures across various phases of the menstrual cycle [14]. Specimens were classified as proliferative (PE, d 8-14), early secretory (ESE, d 15-18), midsecretory (MSE, d 19-23). They did not examine later than Day 23 in the menstrual cycle. Each phase had endometriosis more than one type. We re-analyzed their microarray CEL files and each phase was treated as a separate data set. In proliferative phase, 4 pathways were up-regulated and 3 pathways were down-regulated. Early secretory phase had the most over-represented pathways, 12 were up-regulated and 21 were down-regulated. There is no significant pathway up-regulated at p ≤ 0.05 in midsecretory phase while 22 down-regulated pathways were identified [See Additional file 3]. The overlap of pathways among these phases is very low (Figure 3).

### Differential expression in human endometrial endothelial cells

Sha et al. selected the eutopic endothelial compartment as the subject for exploring the differential expression profile between endometriosis patients and normal controls [4]. Our re-analysis revealed 44 up-regulated and 1 down-regulated pathways in the dataset [See Additional file 4]. There is no overlap compared with the common list from ovarian endometriosis data sets using the whole tissue as sample.

## Discussion

Endometriosis is an enigmatic disease. No existing single theory can explain all cases of endometriosis. The genome-wide microarrays are very powerful because they allow the identification of gene families or pathways that change in concert in a disease state comprehensively. Biologically relevant inference should therefore be reproducible across laboratories. For single gene analysis, different statistical methods and different datasets examining the same biological condition may lead to significant discrepancies [26]. Pathways analysis applied to different datasets yields interesting common results, diminishing the large discrepancies observed in direct comparisons of lists of differentially expressed genes obtained from different datasets. Therefore, the study of complex diseases through pathway analysis is able to highlight genes weakly connected to the phenotype which may be difficult to detect by using classical univariate statistics.

We have performed gene set enrichment analysis of six independent publicly available gene expression data sets to understand in depth the common biological mechanisms involved in endometriosis. Our study compared the gene expression between lesion locations (ovarian vs. peritoneal), phases of the uterine cycle (proliferative to midsecretory) and cell types (endometrial endothelial cells vs. whole tissue), as well as overall eutopic versus ectopic endometrium. The transcriptomes of eutopic endometrium and ectopic endometrial lesions suggest that ovarian endometriosis and peritoneal disease are different disorders [13]. Our findings suggest that most of the pathways impacted the ovarian and peritoneal endometriosis are consistent. Many of differentially expressed pathways found in this study have already been reported to be involved in endometriosis pathogenesis. Here, this discussion presents several of the differentially expressed pathways and hypotheses regarding the role of these pathways in endometriosis.

Most significant of the common up-regulated pathways are involved in immune system and immune disorders. It has been widely documented that endometriosis, as an inflammatory disease, induces an immune response, leading to both cellular and humoral immune changes [27,28]. The association between endometriosis and immune disorders were literature supported [29]. Also some studies concluded that women with endometriosis do not have a higher risk of having asthma, systemic lupus erythematosus and Sjögren's syndrome than other subjects [30,31]. Our GSEA results showed that expression of Asthma, Graft-versus-host disease, Autoimmune thyroid disease, Allograft rejection, Systemic lupus erythematosus and Type I diabetes mellitus pathways are the significantly imbalanced between endometriosis and eutopic endometrium. We found that human leukocyte antigen (HLA) genes are critical genes in these pathways. HLA are key components of the major histocompatibility complex (MHC), which is involved in immune cell signalling processes such as T-cell activation. People with certain HLA antigens are more likely to develop certain autoimmune diseases, such as Type I Diabetes, Ankylosing spondylitis, Celiac Disease, Systemic Lupus Erythematosus, Myasthenia Gravis and Sjögren's syndrome et al [32].

Cytokine-cytokine receptor interaction and Cell adhesion molecules (CAMs) included in GSEA were up-regulated in endometriosis. Cell adhesion molecules are (glyco) proteins expressed on the cell surface and play a critical role in a wide array of biologic processes that include hemostasis, the immune response, inflammation, embryogenesis, and development of neuronal tissue. Clinical observations and in vitro experiments imply that endometriotic cells are invasive and able to metastasize. Analogous to tumour metastasis, it is likely that cell adhesion molecules are central for the invasion and metastasis of endometriotic cells. The expression of some integrins is aberrant in endometriotic lesions compared to eutopic endometrium [33]. Cytokines are key mediators of intercellular communication within the immune system. Several cytokines including interleukin (IL)-1, 6, 8, 10, tumor necrosis factor (TNF)-α, and vascular endothelial growth factor (VEGF) were reported to be increased in the peritoneal fluid (PF) of women with endometriosis [34-41]. Peroxisome proliferator-activated receptors (PPARs) signaling pathway is up-regulated according to GSEA. PPAR are nuclear hormone receptors that are activated by fatty acids and their derivatives. PPAR-γ is present in human ovarian cells. Activation of PPAR-γ enhances steroidogenesis via activation of StAR protein and leads to the activation of insulin-signaling pathways [42].

The expression patterns of ER (estrogen receptors) and PR (progesterone receptors) in endometriotic lesions are different from those in the eutopic endometrium. Endometriosis is an estrogen-dependent disease [43]. Studies of hormone-ligand binding assays and enzyme immunoassays showed a consistent reduction in the content of ER and PR in endometriotic implants [44-47]. Androgen and estrogen metabolism pathway appeared in most of our re-analysis results. Oxidative phosphorylation pathway possibly affect oocyte quality, fertilization rate, and further embryo development [48], is down-regulated in our analysis.

Burney et al. studied proliferative, early secretory and mid-secretory eutopic endometrium (up to Day 23), from women with endometriosis and controls. They found that endometrial gene expression differed most, between these groups, in the early secretory phase (Days 15-18). They found far fewer differences in the mid-secretory phase where no transcripts were found to be up- or down-regulated 4-fold. The result is consistent with the findings of other studies [12,49,50]. Corresponding to their result, there is no significant up-regulated pathway in mid-secretory phase by our GSEA. The molecular phenotype of mid-secretory, eutopic endometrium from women with endometriosis and from controls appears to be very similar [50]. Current efforts to develop minimally invasive diagnostic tests for the presence of endometriosis and also tests to distinguish minimal/mild and moderate/severe disease, by sampling the endometrium, should be focused on the early secretory phase of the menstrual cycle [50].

We hypothesize that all cell types in the endometriotic lesion contribute to the pathology of the disease. Matsuzaki et al. compared global gene expression in eutopic endometrium, from controls and patients with deep endometriosis, at various time points throughout the menstrual cycle. They found no genes were up- or down-regulated in all phases of the cycle, in either tissue compartments [12]. None of the genes from their study that had been identified as differentially expressed in either the stromal or epithelial compartments was shown to be differentially expressed. This may be due to the relative contribution that the epithelial and stromal transcriptomes make to whole tissue gene expression. Our GSEA result showed that the significant pathways from the human endometrial endothelial cells had low overlap with the list from ovarian endometriosis data sets used whole tissue.

## Conclusion

The pathogenesis of endometriosis is likely multifactorial. A deeper understanding of the mechanisms of these diseases can be reached by focusing on deregulation of gene sets or pathways rather than on individual genes. By standardised microarray preprocessing and GSEA, we have increased the concordance to identify many biological mechanisms are involved in endometriosis which are novel in terms of their connection to endometriosis (as mined from the existing literature). More studies about the specific role and interactions of the genes included in related pathways are needed to improve the understanding of endometriosis.

## Competing interests

The authors declare that they have no competing interests.

## Authors' contributions

HBZ and YCP conceived and designed the study. HBZ contributed the data analysis and drafted the manuscript. KH performed data collection. QSW and CYB mined the literature and manuscript drafting. All authors read and approved the final manuscript.

## Supplementary Material

Additional file 1 **Table 5: Pathway analysis of ovarian endometriosis data sets**. The data provided represent the list of significant pathways identified by GSEA in the 3 ovarian endometriosis data sets. Click here for file

Additional file 2 **Table 6: Pathway analysis of peritoneal endometriosis data sets**. The data provided represent the list of significant pathways identified by GSEA in the 2 peritoneal endometriosis data sets. Click here for file

Additional file 3 **Table 7: Pathway analysis between the timing of the uterine cycle**. The data provided represent the list of significant pathways identified by GSEA between the timing of the uterine cycle. Click here for file

Additional file 4 **Table 8: Pathway analysis in the specific phenotype of HEECs from endometriosis**. The data provided represent the list of significant pathways identified by GSEA involved in the specific phenotype of HEECs from endometriosis. Click here for file

Additional file 5 **Table 9: Common significant pathways in ovarian endometriosis data sets**. The data provided represent the list of common significant pathways from GSEA of the 3 ovarian endometriosis datasets. Click here for file

Additional file 6 **Table 10: Common significant pathways in peritoneal endometriosis data sets**. The data provided represent the list of common significant pathways from GSEA of the 2 peritoneal endometriosis datasets. Click here for file

## References

[B1] Eskenazi B, Warner ML (1997). Epidemiology of endometriosis. Obstet Gynecol Clin North Am.

[B2] Giudice LC, Kao LC (2004). Endometriosis. Lancet.

[B3] Bulun SE (2009). Endometriosis. N Engl J Med.

[B4] Sha G, Wu D, Zhang L, Chen X, Lei M, Sun H, Lin S, Lang J (2007). Differentially expressed genes in human endometrial endothelial cells derived from eutopic endometrium of patients with endometriosis compared with those from patients without endometriosis. Hum Reprod.

[B5] Kato N, Sasou S, Motoyama T (2006). Expression of hepatocyte nuclear factor-1beta (HNF-1beta) in clear cell tumors and endometriosis of the ovary. Mod Pathol.

[B6] Eyster KM, Boles AL, Brannian JD, Hansen KA (2002). DNA microarray analysis of gene expression markers of endometriosis. Fertil Steril.

[B7] Lebovic DI, Baldocchi RA, Mueller MD, Taylor RN (2002). Altered expression of a cell-cycle suppressor gene, Tob-1, in endometriotic cells by cDNA array analyses. Fertil Steril.

[B8] Arimoto T, Katagiri T, Oda K, Tsunoda T, Yasugi T, Osuga Y, Yoshikawa H, Nishii O, Yano T, Taketani Y, Nakamura Y (2003). Genome-wide cDNA microarray analysis of gene-expression profiles involved in ovarian endometriosis. Int J Oncol.

[B9] Kao LC, Germeyer A, Tulac S, Lobo S, Yang JP, Taylor RN, Osteen K, Lessey BA, Giudice LC (2003). Expression profiling of endometrium from women with endometriosis reveals candidate genes for disease-based implantation failure and infertility. Endocrinology.

[B10] Fan Y, Chen BL, Ma XD, Su MQ (2005). Detection of expression of endometriosis-related cytokine and their receptor genes by cDNA microarray technique. Xi Bao Yu Fen Zi Mian Yi Xue Za Zhi.

[B11] Matsuzaki S, Canis M, Pouly JL, Dechelotte P, Okamura K, Mage G (2005). The macrophage stimulating protein/RON system: a potential novel target for prevention and treatment of endometriosis. Mol Hum Reprod.

[B12] Matsuzaki S, Canis M, Vaurs-Barriere C, Boespflug-Tanguy O, Dastugue B, Mage G (2005). DNA microarray analysis of gene expression in eutopic endometrium from patients with deep endometriosis using laser capture microdissection. Fertil Steril.

[B13] Wu Y, Kajdacsy-Balla A, Strawn E, Basir Z, Halverson G, Jailwala P, Wang Y, Wang X, Ghosh S, Guo SW (2006). Transcriptional characterizations of differences between eutopic and ectopic endometrium. Endocrinology.

[B14] Burney R, Talbi S, Hamilton A, Vo K, Nyegaard M, Nezhat C, Lessey B, Giudice L (2007). Gene Expression Analysis of Endometrium Reveals Progesterone Resistance and Candidate Susceptibility Genes in Women with Endometriosis. Endocrinology.

[B15] Eyster KM, Klinkova O, Kennedy V, Hansen KA (2007). Whole genome deoxyribonucleic acid microarray analysis of gene expression in ectopic versus eutopic endometrium. Fertil Steril.

[B16] Hever A, Roth R, Hevezi P, Marin M, Acosta J, Acosta H, Rojas J, Herrera R, Grigoriadis D, White E (2007). Human endometriosis is associated with plasma cells and overexpression of B lymphocyte stimulator. Proceedings of the National Academy of Sciences.

[B17] Hull ML, Escareno CR, Godsland JM, Doig JR, Johnson CM, Phillips SC, Smith SK, Tavare S, Print CG, Charnock-Jones DS (2008). Endometrial-peritoneal interactions during endometriotic lesion establishment. Am J Pathol.

[B18] Subramanian A, Tamayo P, Mootha VK, Mukherjee S, Ebert BL, Gillette MA, Paulovich A, Pomeroy SL, Golub TR, Lander ES, Mesirov JP (2005). Gene set enrichment analysis: a knowledge-based approach for interpreting genome-wide expression profiles. Proc Natl Acad Sci USA.

[B19] Mootha VK, Lindgren CM, Eriksson KF, Subramanian A, Sihag S, Lehar J, Puigserver P, Carlsson E, Ridderstrale M, Laurila E, Houstis N, Daly MJ, Patterson N, Mesirov JP, Golub TR, Tamayo P, Spiegelman B, Lander ES, Hirschhorn JN, Altshuler D, Groop LC (2003). PGC-1alpha-responsive genes involved in oxidative phosphorylation are coordinately downregulated in human diabetes. Nat Genet.

[B20] Jing Shi, Walker MG (2007). Gene set enrichment analysis (GSEA) for interpreting gene expression profiles. Current Bioinformatics.

[B21] Gentleman RC, Carey VJ, Bates DM, Bolstad B, Dettling M, Dudoit S, Ellis B, Gautier L, Ge Y, Gentry J, Hornik K, Hothorn T, Huber W, Iacus S, Irizarry R, Leisch F, Li C, Maechler M, Rossini AJ, Sawitzki G, Smith C, Smyth G, Tierney L, Yang JY, Zhang J (2004). Bioconductor: open software development for computational biology and bioinformatics. Genome Biol.

[B22] R Development Core Team (2009). R: A language and environment for statistical computing.

[B23] Irizarry RA, Hobbs B, Collin F, Beazer-Barclay YD, Antonellis KJ, Scherf U, Speed TP (2003). Exploration, normalization, and summaries of high density oligonucleotide array probe level data. Biostatistics.

[B24] Gautier L, Cope L, Bolstad BM, Irizarry RA (2004). affy--analysis of Affymetrix GeneChip data at the probe level. Bioinformatics.

[B25] (2009). Using Categories to Model Genomic Data. http://www.bioconductor.org/packages/2.4/bioc/vignettes/Category/inst/doc/Category.pdf.

[B26] Manoli T, Gretz N, Grone H-J, Kenzelmann M, Eils R, Brors B (2006). Group testing for pathway analysis improves comparability of different microarray datasets. Bioinformatics.

[B27] Sinaii N, Cleary SD, Ballweg ML, Nieman LK, Stratton P (2002). High rates of autoimmune and endocrine disorders, fibromyalgia, chronic fatigue syndrome and atopic diseases among women with endometriosis: a survey analysis. Hum Reprod.

[B28] Lebovic DI, Mueller MD, Taylor RN (2001). Immunobiology of endometriosis. Fertility and Sterility.

[B29] Berbic M, Schulke L, Markham R, Tokushige N, Russell P, Fraser IS (2009). Macrophage expression in endometrium of women with and without endometriosis. Hum Reprod.

[B30] Ferrero S, Petrera P, Colombo BM, Navaratnarajah R, Parisi M, Anserini P, Remorgida V, Ragni N (2005). Asthma in women with endometriosis. Hum Reprod.

[B31] Matorras R, Ocerin I, Unamuno M, Nieto A, Peiro E, Burgos J, Exposito A (2007). Prevalence of endometriosis in women with systemic lupus erythematosus and Sjogren's syndrome. Lupus.

[B32] de Bakker PIW, McVean G, Sabeti PC, Miretti MM, Green T, Marchini J, Ke XY, Monsuur AJ, Whittaker P, Delgado M, Morrison J, Richardson A, Walsh EC, Gao XJ, Galver L, Hart J, Hafler DA, Pericak-Vance M, Todd JA, Daly MJ, Trowsdale J, Wijmenga C, Vyse TJ, Beck S, Murray SS, Carrington M, Gregory S, Deloukas P, Rioux JD (2006). A high-resolution HLA and SNP haplotype map for disease association studies in the extended human MHC. Nature Genet.

[B33] Starzinski-Powitz A, Handrow-Metzmacher H, Kotzian S (1999). The putative role of cell adhesion molecules in endometriosis: can we learn from tumour metastasis?. Molecular Medicine Today.

[B34] Wu MY, Ho HN, Chen SU, Chao KH, Chen CD, Yang YS (1999). Increase in the production of interleukin-6, interleukin-10, and interleukin-12 by lipopolysaccharide-stimulated peritoneal macrophages from women with endometriosis. Am J Reprod Immunol.

[B35] Ho HN, Wu MY, Chao KH, Chen CD, Chen SU, Yang YS (1997). Peritoneal interleukin-10 increases with decrease in activated CD4+ T lymphocytes in women with endometriosis. Hum Reprod.

[B36] Ho HN, Wu MY, Chao KH, Chen CD, Chen SU, Chen HF, Yang YS (1996). Decrease in interferon gamma production and impairment of T-lymphocyte proliferation in peritoneal fluid of women with endometriosis. Am J Obstet Gynecol.

[B37] Keenan JA, Chen TT, Chadwell NL, Torry DS, Caudle MR (1995). IL-1 beta, TNF-alpha, and IL-2 in peritoneal fluid and macrophage-conditioned media of women with endometriosis. Am J Reprod Immunol.

[B38] Harada T, Yoshioka H, Yoshida S, Iwabe T, Onohara Y, Tanikawa M, Terakawa N (1997). Increased interleukin-6 levels in peritoneal fluid of infertile patients with active endometriosis. Am J Obstet Gynecol.

[B39] Punnonen J, Teisala K, Ranta H, Bennett B, Punnonen R (1996). Increased levels of interleukin-6 and interleukin-10 in the peritoneal fluid of patients with endometriosis. Am J Obstet Gynecol.

[B40] Hornung D, Bentzien F, Wallwiener D, Kiesel L, Taylor RN (2001). Chemokine bioactivity of RANTES in endometriotic and normal endometrial stromal cells and peritoneal fluid. Mol Hum Reprod.

[B41] Ponce C, Torres M, Galleguillos C, Sovino H, Boric MA, Fuentes A, Johnson MC (2009). Nuclear Factor kappa B (NF{kappa}B) pathway and Interleukin-6 (IL-6) are affected in eutopic endometrium of women with endometriosis. Reproduction.

[B42] Seto-Young D, Avtanski D, Strizhevsky M, Parikh G, Patel P, Kaplun J, Holcomb K, Rosenwaks Z, Poretsky L (2007). Interactions among Peroxisome Proliferator Activated Receptor-{gamma}, Insulin Signaling Pathways, and Steroidogenic Acute Regulatory Protein in Human Ovarian Cells. J Clin Endocrinol Metab.

[B43] Bulun SE, Yang S, Fang Z, Gurates B, Tamura M, Sebastian S (2002). Estrogen production and metabolism in endometriosis. Ann N Y Acad Sci.

[B44] Tamaya T, Motoyama T, Ohono Y, Ide N, Tsurusaki T, Okada H (1979). Steroid receptor levels and histology of endometriosis and adenomyosis. Fertil Steril.

[B45] Janne O, Kauppila A, Kokko E, Lantto T, Ronnberg L, Vihko R (1981). Estrogen and progestin receptors in endometriosis lesions: comparison with endometrial tissue. Am J Obstet Gynecol.

[B46] Lyndrup J, Thorpe S, Glenthoj A, Obel E, Sele V (1987). Altered progesterone/estrogen receptor ratios in endometriosis. A comparative study of steroid receptors and morphology in endometriosis and endometrium. Acta Obstet Gynecol Scand.

[B47] Bergqvist A, Ferno M (1993). Estrogen and progesterone receptors in endometriotic tissue and endometrium: comparison according to localization and recurrence. Fertil Steril.

[B48] Yamashita Y, Asano M, Morishima S, Fujino K, Terai Y, Ohmichi M (2007). Mitochondrial gene expression in granulosa cells of severe endometriosis with in vitro fertilization and embryo transfer. Fertil Steril.

[B49] Kao LC, Germeyer A, Tulac S, Lobo S, Yang JP, Taylor RN, Osteen K, Lessey BA, Giudice LC (2003). Expression Profiling of Endometrium from Women with Endometriosis Reveals Candidate Genes for Disease-Based Implantation Failure and Infertility. Endocrinology.

[B50] Sherwin JRA, Sharkey AM, Mihalyi A, Simsa P, Catalano RD, D'Hooghe TM (2008). Global gene analysis of late secretory phase, eutopic endometrium does not provide the basis for a minimally invasive test of endometriosis. Hum Reprod.

